# Perceived and Real Aquatic Competence in Children from 6 to 10 Years Old

**DOI:** 10.3390/ijerph17176101

**Published:** 2020-08-21

**Authors:** Aldo M. Costa, Alexandra Frias, Sandra S. Ferreira, Mario J. Costa, António J. Silva, Nuno D. Garrido

**Affiliations:** 1 Department of Sport Sciences, University of Beira Interior, 6201-001 Covilhã, Portugal; alexandra.castro@gmail.com; 2 Research Center in Sport Sciences, Health Sciences and Human Development, CIDESD, 5000-801 Vila Real, Portugal; mario.costa@ipg.pt (M.J.C.); ajsilva@utad.pt (A.J.S.); ngarrido@utad.pt (N.D.G.); 3 Health Sciences Investigation Center, University of Beira Interior, CICS-UBI, 6201-001 Covilhã, Portugal; 4 Department of Mathematics, University of Beira Interior, 6201-001 Covilhã, Portugal; sandraf@ubi.pt; 5 Center of Mathematics and Applications, University of Beira Interior, 6201-001 Covilhã, Portugal; 6 Department of Sport Sciences, Polytechnic Institute of Guarda, 6300-035 Guarda, Portugal; 7 Department of Sport Sciences, Exercise and Health, University of Trás-os-Montes and Alto Douro, 5000-801 Vila Real, Portugal

**Keywords:** aquatic skills, children, perceived competence, water safety, drowning prevention

## Abstract

This study aimed to analyze the relationship between perceived aquatic competence (PAC) and real aquatic competence (RAC) in 6 to 10 year old children in skills identified as relevant for surviving an aquatic accident. The study sample consisted of 105 children (8.2 + 1.3 years old). Two age groups were examined separately [G1, 6 to 7 years (*n* = 53); G2, 8 to 10 years (*n* = 52)]. Children’s aquatic competence was evaluated twice for skills linked to the risk of drowning: Firstly, using a common swimsuit (simple condition) and secondly wearing a t-shirt (complex condition). The PAC was assessed by questionnaire interview. Pearson correlation coefficients, pairwise, and independent *t*-test comparisons were performed with a significance level of 5%. Similar levels of PAC were found among both age groups for all measured skills, excepted for breath control during swimming (*p* < 0.05). However, PAC differs significantly (*p* < 0.001) from RAC (in simple and complex conditions) only in G1. Correlations between PAC and RAC were modest for all measured skills in both age-groups. Significant differences were found between RAC in simple and complex conditions in both groups (*p* < 0.01). Age contributes to a higher perceived aquatic competence in skills related to the risk of drowning.

## 1. Introduction

Accidents have been reported as the principal cause of death in children [1], with pedestrian behaviour [2,3,4] and drowning [5,6,7] taking higher attention. In most of the drowning or near-drowning episodes, victims have a history of inadequate or insufficient aquatic skills for safety [8] which includes the inability to displace themselves into a safer zone, change body position, and swim in open water [9].

Regardless of the current sensitivity to drowning prevention, swimming teaching remains focused more on the production of gestures than on the production of solutions [10], challenging the question that learning to swim is a dynamic process in which the person, the proposed tasks, and the context all influence the learning process and the outcome [11], highlighting that if not, almost all swimming classes are taught in a stable and predictable context [12]. As swimming activity (as well as drowning) are both dynamic, changes in the individual characteristics, in the task at hand, or even in the context (e.g., water conditions), can drastically and rapidly change the “ability to swim” [13].

Despite the drowning causes not being well established, aquatic competence arises as one of the most important aspects of survival, mostly in unknown and unstable aquatic environments [14,15]. Langendorfer and Bruya [16] introduced the concept of aquatic competence, representing a state of aquatic proficiency that reduces the risk of drowning and increases the ability to perform tasks in aquatic environments successfully. Without specifically referring to the ability to swim any stroke at any given distance, Langendorfer [13] presented a more comprehensive concept, which was then adapted by Moran et al. [17] to drowning prevention as “the sum of all personal aquatic movements that help prevent drowning, as well as the associated water safety knowledge, attitudes, and behavior that facilitate safety in, on and around the water”, integrating both cognitive and afferent competencies. This integration distinguishes the concept of aquatic competence as especially relevant in the context of drowning prevention, and because of that, it should be considered a dynamic concept as well [18].

Conceptually, competence is defined as the ability to show output in a pre-determined context [19]. The development of several skills and knowledge in life is a pre-requisite to demonstrate efficacy in a singular action [20]. Zarifian [21] defines competence as the practical intelligence supported in solid knowledge, which changes depending on the context complexity. Thus, children show competence when they are able to interpret and demonstrate the most adequate behaviour in a singular situation [22].

According to the nature of the output, competence can be classified as [23]: (i) Cognitive (while showing intellectual and academic skills); (ii) social (based in communication and relationship with peers); and (iii) physical (supported in the game cooperation and sports performance). This rating of competence is extremely important mostly because children, and even many adults, are not capable of having the most accurate perception of their ability in different actions [22], whereas perception represents the individual sense about the ability to conduct a determined task being influenced by some personal traits and the complexity of the event [19,22].

At an early age, children acquire motor knowledge and perceive motor ability in educational or group contexts. A large amount of information and situations can lead to episodes of divergence between real and perceived motor competence [19,22]. The interpretation of success in motor tasks during infancy produces intrinsic satisfaction and boosts persistence [24]. Previous studies showed a strong association between perceived competence and intrinsic motivation e.g., [25]. That is the reason why the initial motor experience is extremely important while developing action patterns that will support more complex behaviours later in life e.g., [26].

While interviewing 112 children 3–8 years old, Coppens [27] showed that the ability to distinguish between safe and unsafe situations preceded the ability to specify preventive measures. The author also reported that comprehension about safety and prevention are linked to the highest levels of logic and more complex cognition, which may explain a higher number of accidents in preschool children. In fact, Harter [23] showed that before the age of 8, no children are capable of making specific judgments by themselves. For instance, regarding water safety education, Moran and Gilmore [28] reported that children aged 8–9 presented a good understanding of the hazards at the beach, despite suggesting that some children might have more difficulty to find ways to keep safe at the beach than at swimming pools, rivers, or lakes.

Aquatic competence perception has been studied in recent years. Some have studied the importance caregivers and children (11 years) attribute to swimming [29]; others studied only parental perceptions regarding children (2–4 years) water safety, swimming ability, and swimming lessons [30]. Both studies considered parents/caregivers beliefs to be far different from expert opinion and suggested processes to best inform them about some misconceptions related to swimming and swimming lessons.

Overestimation is usually reported when assessing perceived aquatic competence (PAC) among minority groups [31], or among young adults [17,32,33,34,35]. Only Rejman et al. [6] reported an underestimation of the swimming skills of boys (14–15 years), despite both boys and girls having a poor level of aquatic competencies and needing a more comprehensive aquatic education.

Further on, regarding young adults (university students), Moran et al. [17] compared the perceived and real aquatic competencies as well as the perceived risk of drowning. The abilities were distance, flotation, and swimming underwater, in which participants were unable to predict their skills. This last study provided insight into the differences between what people think they can do and what they can really do. However, accidents involving drowning possibilities pose challenging situations like wearing clothes [33,36], wearing lifejackets [35], or even in a simple task like getting out of the water after such an episode [37]. Unfortunately, in all of the last studies, aquatic competency or the perceived exertion to accomplish the demanded task were overestimated, although the participants were all young adults.

So, swimming instructors should be aware of this problem and act accordingly. Besides the regular teaching of the four swimming strokes, there is a chance to add some aquatic skills in swimming programs to prevent and/or reduce drowning episodes [9]. It is also useful to recognize aquatic competence and understand which skills can be requested in a safer and controlled place (e.g., traditional swimming pool), or in a more aggressive and unknown context using clothes (e.g., open water with light clothes) [29].

To the best of our knowledge, studies on the perception of aquatic competence have been focusing on toddlers, adolescents, and adults, or young adults. The present study concentrates on children aged 6–10 years old trying to fil the gap surrounding aquatic competence in this age group.

The aim of this study was to: (i) Analyze the differences between PAC and real aquatic competence (RAC) considering the children’s age; and (ii) identify the differences in RAC when comparing two exposures (normal swimsuit vs. normal swimsuit plus clothes). It was hypothesized that a less realistic perception would be observed among younger children and in more complex conditions of practice (with clothing).

## 2. Materials and Methods

### 2.1. Participants

The study comprised 105 children from 6 to 10 years (49 girls and 56 boys; 8.2 ± 1.3 years old). This represented a convenience sample gathering children from proximal aquatic facilities, which allowed easy and rigorous access to the present research evaluations. All the children were attending normal swimming classes at public or private swimming pools, twice a week with a class duration of 45 min. According to the aims of the present study, and for data treatment, the sample was divided into two groups defined by age thresholds: (i) From 6 to 7 years (*n* = 53) and (ii) from 8 to 10 years (*n* = 52). The previous experience of the participants was not used as an inclusion criterion. However, children were excluded based on the inability to perform autonomous aquatic displacement at a minimum distance of 12.5 m allowing to create a minimal inclusion standard and classify the children as “able to swim”, showing propulsive mechanisms in the water with breath control and without stops. All procedures were in accordance with the Declaration of Helsinki concerning Human research. The University Ethics Committee approved the experimental procedures with protocol n. ° 92/2018. All parents and/or legal guardians gave their informed written consent for children participating in the study.

### 2.2. Assessment of the Perceived Aquatic Competence

The PAC was assessed using a structured interview conducted by the principal investigator. Each child answered, in random order, a set of pre-defined questions within limited categories. The time of the interview was strictly controlled using the interview guide and following the same pattern with no diversions. Each child spent approximately 8 min answering all questions. The interview guide was shown to each child in a text format and figures, with several answering options (e.g., Likert Scale) as previously described elsewhere [22]. It comprised 10 questions about PAC related to eight aquatic skills identified as important to prevent drowning episodes as proposed by Stallman et al. [9]: *Sk1*—entry (jump or diving) in deep water; *Sk2*—after immersion, recover to the water surface, get alignment, and swim; *Sk3*—dive from the swimming pool edge and swim underwater; *Sk4*—know at least in a rudimentary sense the front crawl and backstroke; *Sk5*—controlled and relaxed breathing; *Sk6*—change body position (from dorsal to ventral and vice-versa); *Sk7*—change swimming direction (turn left or right at front crawl and backstroke); *Sk8*—floating (stop and rest with minimum or without motion). For each aquatic skill, a gradual scale of mastery was defined based on the suggestions of Langendorfer and Bruya [16]. The scale (from 3 to 5 levels of mastery) was previously defined by five experts in the domain of swimming teaching. The levels of mastery were presented by letters and symbols instead of numbers, in order to avoid influencing the children’s response. Critical components of each level were addressed during the interview to increase clarity. A provisional version of the interview guide was tested in a sample of 20 children, for text and images clarity. After considering the smooth changes proposed, the final version was developed as dissected in Appendix A.

### 2.3. Assessment of Real Aquatic Competence

The eight aquatic skills identified as important to prevent drowning episodes were also tested in the water using the “circuit mode”, as proposed by Junge et al. [38], within a normal swimming class. This allowed testing several skills at once, optimizing the time spent in motor actions [14] while having a positive climate. The circuit was prepared in an indoor short course swimming pool, with a minimum and maximum depth of 1.40 m and 2 m, respectively. The aquatic skills were grouped in two as: *group 1—Sk3, Sk7a, Sk7b; group 2—Sk1, Sk2, Sk4a, Sk4b, Sk5, Sk6, Sk8.* Each child performed the full circuit on two distinct occasions 72 h apart: (i) Using the traditional swimsuit with goggles and a swim cap (simple condition); (ii) adding a cotton t-shirt to the traditional swimsuit, without goggles and the swim cap (complex condition). In both sessions, no changes were observed in the water characteristics (pH—7.2; chlorine—1.89 mg/L and temperature ~ 29.0 °C) or air humidity (~50%).

Prior to testing and after a demonstration given by the swimming instructor, children were allowed to try the aquatic skills and its sequence. Researchers also tested reliability while scoring the aquatic skills in a group of eight children not included in the sample. These children performed the test twice in different class days in the same week. The intra-observer reliability was higher than 0.8 for all scoring, suggesting a high level of agreement. In real testing, each child completed the circuit two times with a 5 min interval. The swimming teacher proceeded with the normal class while the skills were assessed by the expert researchers using a pre-defined scoring list. The best score from the two attempts in each skill was used for further analysis.

Table 1 presents the criteria used to assess RAC according to the standards proposed by Stallman et al. [9]. Critical components were additionally added to clarify the assessment. The RAC was converted in a scoring scale, similar to the one described by Langendorfer and Bruya [16] to evaluate aquatic readiness and used in previous studies e.g., [39,40]. There was a high level of coherence between the interview guide and the table description. The table also shows a high level of intra-observer reliability (Cohen’s Kappa > 0.7) for all the aquatic skills tested.

### 2.4. Statistical Analysis

A Windows SPSS^®^ version 23 (IBM Corp., Armonk, NY, USA) was used to compute the data. The sample was divided into two groups. Data were expressed as mean and standard deviation for each skill. Kolmogorov–Smirnov and the Levene tests were used to assess normality and homoscedasticity assumptions, respectively. Since the normal distribution was verified, a student’s *t*-test was computed to assess differences between conditions (PAC vs. RAC; RAC in simple vs. complex condition). Despite the best score being used in each measured skill, the intra-observer reliability was tested using Cohen’s kappa coefficient. The level of statistical significance was set at *p* ≤ 0.05.

## 3. Results

Table 2 presents the differences between PAC and RAC (in simple and complex conditions) in both age groups. Most of the skills tested revealed significant differences between PAC and RAC (simple plus complex) in both age groups. The only exceptions were the *Sk1* and *Sk8* skills. The differences between PAC and RAC were most notorious in the youngest group. Significant differences were found between RAC in simple and complex conditions in the *Sk1*, *Sk3,* and *Sk4a* skills for the youngest group (6–7 years) and in the *Sk2*, *Sk4a*, and *Sk7b* skills for the oldest group (8–10 years). As expected, the younger group exhibited lower overall scores of RAC in both simple and complex conditions.

## 4. Discussion

The aim of this study was to analyze the differences between PAC and RAC in children from 6 to 10 years and compare RAC using traditional swimsuit or adding clothes. The main results suggest that children from 6 to 10 years have a wrong perception about their RAC, and even more so when some constraints, like clothes, are added to a given task. Despite that, older children (8–10 years) exhibited a higher RAC and a minor difference between PAC and RAC, which may reduce their risk of drowning.

Perceived aquatic competence was higher than RAC in both conditions of exposure but more noticeable in the youngest group, suggesting that younger children were more likely to distort, in a higher range, their RAC. Indeed, Murcia and Pérez [22] reported that younger children present a worse perception of learning skills and demonstrate a minor positive attitude in contact with water. According to Zaichkowsky et al. [41], this can be explained by reduced muscular development and motor memory observed at early ages, which may dictate the lower motivation in perceiving their real competence. This issue was already observed and explained in children with diagnosed problems in development and motor coordination [22,42].

Unfortunately, our data were not strong enough to understand the real influence of the previous aquatic experience (weekly frequency and hours of swimming sessions) on the PAC, considering the age effect. One might argue there would be that age effect, as previous studies suggested age as a risk factor when analyzing the causes of death in victims from drowning [9,39,43,44].

Both groups presented a higher PAC in the *Sk4a, Sk5, Sk7a,* and *Sk7b* skills than their RAC in simple and complex conditions. *Sk4a* and *Sk5* skills are mainly focused on the ventral swim. The misjudgment about RAC in those skills sets up a greater risk, even more so when the distance to a secure place increases [9]. Ventral swim, specifically the *Sk4a* skill (“Know at least in a rudimentary sense the front crawl”), sets up several possibilities regarding drowning prevention. It is considered the faster and the most economic technique [45], and when swum head up, enables better all-round visibility permitting one to avoid perils and to select the safer direction [46]. Despite the utilization of other techniques being more favorable in certain contexts, front crawl has been used to test several abilities regarding drowning prevention [32,33,34,35]. In fact, in the Moran Study, some participants struggled to swim front crawl with clothes on. It is important to note that in that study, participants wore, besides a swimsuit and t-shirt, sweatshirt and cotton trousers. Probably, the ability to swim front crawl would be best necessary when having to move fast for a short distance, as when distance increases, techniques other than front crawl become more effective, namely breaststroke [47].

Effective breathing should be comfortable and economic, well-integrated with arms and legs movements, coordinated with balance and body alignment, and most importantly, must embrace the task, the person, and the context [46]. Considering these points, one cannot dissociate the *Sk5* from the other skills as they are strictly connected to the concept of drowning posed by Bierens [48] as a complex and multifaceted phenomenon characterized by a chain of events. In this regard, one cannot forget that drowning is considered a respiratory impairment from immersion or submersion in a liquid [49,50] and that drowning occurs due to failure to breathe at need [46].

The *Sk7a* and *Sk7b* skills are associated with changing directions using the transversal and longitudinal axis of rotation. As in ventral and dorsal swimming positions, they encompass several advantages such as changing direction when the context requires. These are skills that demand higher motor coordination in synchronizing balance, breathing, arms, and legs actions, which may require to reach a higher motor development stage, to take in the most effective and efficient swimming pattern. Nevertheless, Asher et al. [51] with toddlers and Oliveira et al. [52] with five-year-old children, reported the ability of children to roll over and turn. Moreover, those skills are included further in swimming programs and may require more aquatic experience to be well perceived and mastered.

The youngest group showed a lower PAC in the *Sk2* skill (“after immersion, recover to the water surface, get alignment and swim”) and an even lower RAC. Even though the causes of drowning are not well established in the literature, Golden and Tipton [53] report that most of the accidents begin with a fall in the water at a minor distance from a secure place. Swimming programs worldwide are not rigorously structured to teach how to behave when falling in the water, and how to find or reach safer places after that. Moreover, most programs focus on the traditional swim tasks instead of adding unexpected challenges like working with depth of the fall, and the fall distance from the wall, or the exiting from the water [35,37]. So, these findings attribute greater importance to what children may perceive and execute in the *Sk2* skill as this is an aspect that may solve unexpected falls both in swimming pool and in the open water context.

This study also aimed to understand PAC and RAC in two conditions of exposure (in the normal swimsuit or adding clothes). As expected, some distortion about PAC and RAC was shown in 4 of the 10 measured skills in both age groups, namely *Sk4a*, *Sk5, Sk7a,* and *b*; in *Sk2* and *Sk3* for the 6–7-year-old group and in *Sk4b* and *Sk6* for the 8–10-year-old group. This result highlights the dynamic concept of aquatic competence proposed by Langendorfer [13], adapted from the ecological model of Newell [54], in which the individual, the task, and the context influence the teaching-learning process in an integrated way and, inherently, the final product of the pedagogical action (acquired aquatic competence). Indeed, the development of aquatic safety should not be considered incompatible or inconsistent with the more conservative objectives of swimming education programs [55]. The variability of the teaching contexts (e.g., depth, water temperature, salinity, water opacity, current, types of installation) and the conditions of execution (e.g., equipment used, task objectives), could be, from our point of view, an added pedagogical value to swimming teaching programs. Any initiative to develop the aquatic motor repertoire in varied aquatic contexts, associated with the incentive to improve knowledge, attitudes, and behaviors that facilitate safety in the aquatic environment, is, by definition [32], drowning prevention.

Some limitations should be addressed to this study: (i) The absence of concrete data (weekly frequency and total hours of exposure) about the previous experience in swimming programs, which impaired the ability to see the real effect in perceived and real aquatic competence; (ii) although the content validity of the questionnaire was performed, researchers and practitioners should have some caution when using it in further studies; (iii) some water competencies recently reported as determinants for water safety (use of personal flotation devices and open water competencies) were not included at this point and should be added in the future for a complete guide to measure PAC and RAC; (iv) even though wearing a t-shirt provided additional difficulty to the children it will never resample a real open water situation or accident episode, where most likely, people would be wearing full clothes; (v) testing was made in a controlled and warm environment where children are used to having swimming classes. Besides testing and the t-shirt, the common routines were implemented by the swimming instructor, and none of the normal characteristics like air and water temperature or water opacity were changed. All of these confounding factors could have changed the present results and should be considered in future research.

## 5. Conclusions

Our results have shown that perceived aquatic competence differed significantly from real aquatic competence in most of the skills identified by the literature as important for drowning prevention. Younger children are more likely to overestimate their actual aquatic competence, especially if evaluated under more complex conditions (with clothing). The scoring scale for aquatic competence tends to be lower in complex conditions, especially in skills related to deep water entry/dive, underwater swimming, immersion, rudimentary swimming, and changing swimming direction. Overestimating aquatic competence could endanger this population, which is not used to swimming outside the comfort of the swimming classes and with clothes. In order to avoid becoming at risk, parents or caregivers should be advised concerning the present results, as no aquatic competence replaces the supervision, mostly at these ages.

## Figures and Tables

**Table 1:** Scoring scale to evaluate aquatic competence related to the risk of drowning.

Skill	Scoring Scale	*K*	
		Simple Condition	Complex Condition
(*Sk1*) Entry (jump or diving) in deep water	0. Unable to enter the water voluntarily 1. Performs a foot entry with assistance 2. Performs a foot entry without being assisted 3. Performs a head diving with assistance 4. Performs a head diving without assistance	1.0	0.932
(*Sk2*) After immersion, recover to the water surface, get alignment and swim	0. Unable to regain horizontality or start swimming 1. Unable to regain horizontality but starts swimming 2. Partially recovers horizontality and starts swimming 3. Regain horizontality and start swimming	0.919	0.955
(*Sk3*) Dive from the swimming pool edge and swim underwater	0. Unable to jump into the water 1. Performs a jump into the water but is not able to perform an underwater course. 2. Performs a jump into the water followed by a short underwater course (<2 m) 3. Performs a jump into the water followed by a medium underwater course (<3 m) 4. Performs a jump into the water followed by a long underwater course (>3 m)	0.726	0.749
(*Sk4a*) Know at least in a rudimentary set the front crawl	0. Does not reveal any propulsive motor behavior in the ventral position 1. Performs rudimentary swimming (globally known as “locomotive swimming”) 2. The beginning of the formal stroke is identified 3. Performs a rudimentary swimming technique (breathing, body position, kick, and arm strokes) 4. Performs an advanced swimming technique	0.870	0.950
(*Sk4b*) Know at least in a rudimentary set the backstroke	0. There is no propulsive motor behavior in the dorsal position 1. Performs an undefined propulsive movement in the upright or seated dorsal position 2. The beginning of the formal stroke is identified 3. Performs a rudimentary swimming technique (breathing, body position, kick, and arm strokes) 4. Performs an advanced swimming technique	0.957	0.956
(*Sk5*) Controlled and relaxed breathing	0. Performs a respiratory blockage during swimming 1. Performs respiratory cycles but without synchronization with the action of arms and legs 2. Performs respiratory rhythms synchronized with swimming but with loss of body alignment 3. Performs rhythmic breathing cycles, synchronized with swimming and with correct body alignment	0.944	0.934
(*Sk6*) Change body position (from dorsal to ventral and vice-versa)	0. Unable to perform rotation 1. Performs the rotation in the longitudinal axis, but with loss of body horizontality 2. Performs the rotation in the longitudinal axis without loss of the body horizontality	0.961	0.962
(*Sk7a*) Change swimming direction (turn left or right at front crawl)	0. Unable to change direction during swimming 1. Performs swimming direction but with successive stops 2. Performs swimming direction changes without interruption	0.781	0.847
(*Sk7b*) Change swimming direction (turn left or right at backstroke)	0. Unable to change direction during swimming 1. Performs swimming direction but with successive stops 2. Performs swimming direction changes without interruption	0.772	0.891
(*Sk8*) Floating (stop and rest with minimum or without motion)	0. Unable to maintain static vertical balance 1. Maintain static vertical balance, but with pronounced propulsive movements 2. Maintain static vertical balance without sharp propulsive movements	0.903	0.948

Legend: K—intra-observer reliability (Cohen’s Kappa coefficient).

**Table 2:** Perceived and real aquatic competence (mean ± SD) for all the skills tested in both groups.

Skill	Age Group	Perceived Aquatic Competence		Student’s *t*-Test (″)			Real Aquatic Competence (Simple)		Student’s *t*-Test (□)			Real Aquatic Competence (Complex)		Student’s *t*-Test (ϕ)		
				Differencein Means	t	*p*			Differencein Means	t	*p*			Differencein means	t	*p*
*Sk1 (0–4 pts.)*	6–7	3.74	±0.59	0.01887	0.139	0.890	3.72	±0.69 ^ϕ^	0.16981	1.219	0.228	3.57	±0.82	0.15094	2.060	0.044
	8–10	3.79	±0.67	0.05769	0.553	0.582	3.73	±0.72	−0.01923	−0.240	0.811	3.81	±0.69	−0.07692	−1.272	0.209
*Sk2 (0–3 pts.)*	6–7	2.17	±0.73 ^□^	0.28302	1.938	0.058	1.89	±0.61	0.43396	2.825	0.007	1.74	±0.74	0.15094	1.531	0.132
	8–10	2.29	±0.72	0.05769	0.444	0.659	2.23	±0.67 ^ϕ^	0.25000	1.756	0.085	2.04	±0.71	0.19231	2.210	0.032
*Sk3 (0–4 pts.)*	6–7	3.28	±0.86 ^″^	−0.32075	−2.442	0.018	3.60	±0.77 ^ϕ^	0.05660	0.302	0.764	3.23	±1.10	0.37736	3.195	0.002
	8–10	3.54	±0.67	−0.19231	−1.749	0.086	3.73	±0.69	−0.13462	−1.000	0.322	3.67	±0.88	0.05769	0.622	0.537
*Sk4a (0–4 pts.)*	6–7	3.36	±0.74 ^″;□^	0.90566	5.743	0.000	2.45	±0.95 ^ϕ^	1.24528	7.228	0.000	2.11	±1.10	0.33962	2.691	0.010
	8–10	3.52	±0.58 ^″;□^	0.42308	3.001	0.004	3.10	±0.98	0.51923	3.369	0.001	3.00	±1.07	0.09615	1.527	0.133
*Sk4b (0–4 pts.)*	6–7	3.62	±0.74 ^″;□^	0.83019	6.937	0.000	2.79	±0.74	0.88679	6.044	0.000	2.74	±0.88	0.05660	0.724	0.472
	8–10	3.69	±0.61 ^□^	0.19231	1.322	0.192	3.50	±0.78 ^ϕ^	0.42308	3.001	0.004	3.27	±0.74	0.23077	2.863	0.006
*Sk5 (0–3 pts.)*	6–7	2.04	±0.98 ^″;□^	0.50943	4.067	0.000	1.53	±0.85	0.60377	4.452	0.000	1.43	±1.01	0.09434	1.093	0.279
	8–10	2.46	±0.83 ^″;□^	0.28846	2.329	0.024	2.17	±0.79	0.38462	2.976	0.004	2.08	±0.90	0.09615	1.093	0.279
*Sk6 (0–2 pts.)*	6–7	1.43	±0.75	−0.05660	−0.574	0.569	1.49	±0.50	−0.01887	−0.172	0.864	1.45	±0.50	0.03774	0.629	0.532
	8–10	1.50	±0.64 ^″^	−0.23077	−2.579	0.013	1.73	±0.49	−0.15385	−1.592	0.118	1.65	±0.52	0.07692	1.660	0.103
*Sk7a (0–2 pts.)*	6–7	1.74	±0.45 ^″;□^	−0.20755	−3.328	0.002	1.94	±0.23	−0.20755	−3.055	0.004	1.94	0.23	0.00000	0.000	1.000
	8–10	1.63	±0.49 ^″;□^	−0.36538	−5.419	0.000	2.00	±0.00	−0.30769	−4.081	0.000	1.94	0.24	0.05769	1.767	0.083
*Sk7b (0–2 pts.)*	6–7	1.51	±0.58 ^″;□^	−0.32075	−3.470	0.001	1.83	±0.43	−0.30189	−3.444	0.001	1.81	±0.44	0.01887	0.375	0.709
	8–10	1.38	±0.53 ^″;□^	−0.61538	−8.378	0.000	2.00	±0.00 ^ϕ^	−0.50000	−5.150	0.000	1.88	±0.32	0.11538	2.579	0.013
*Sk8 (0–2 pts.)*	6–7	1.34	±0.68	0.03774	0.362	0.719	1.30	±0.67	0.11321	1.000	0.322	1.23	±0.58	0.07547	1.000	0.322
	8–10	1.40	±0.60	−0.01923	−0.184	0.855	1.42	±0.64	−0.07692	−0.753	0.455	1.48	±0.61	−0.05769	−1.000	0.322
*Total (%)*	6–7	80.75	±11.37 ^″;□^	1.67925	3.186	0.002	75.16	±12.58 ^ϕ^	2.98113	4.558	0.000	70.82	±15.66	1.30189	4.347	0.000
	8–10	84.04	±9.01	−0.40385	−0.815	0.419	85.38	±13.25 ^ϕ^	0.38462	0.682	0.499	82.76	±14.90	0.78846	3.126	0.003

Legend: (pts.) points interval of each skill evaluated; (″) significant between PAC and RAC (simple); (□) significant between PAC and RAC (complex); (ϕ) significant between RAC (simple) and RAC (complex).

## Appendix A

### Assessment of the Perceived Aquatic Competence (Structured Interview)

(*Sk1*) Can you jump into the water?				

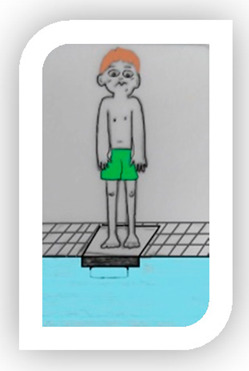
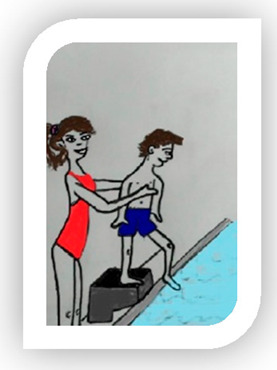
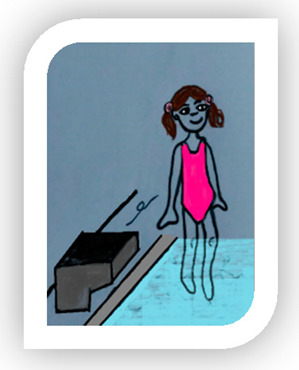
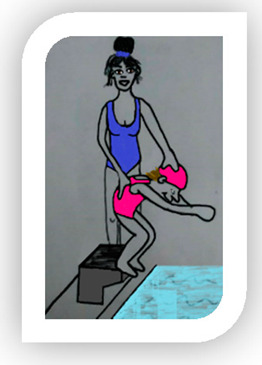
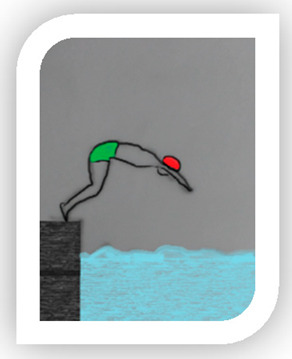
				
A. “I cannot jump into the water”	B. “I can perform a foot entry, but I need the teacher assistance”	C. “I can perform a foot entry all by myself”	D. “I can perform a head diving, but I need the teacher assistance”	E. “I can perform a head diving all by myself”

(*Sk2*) In deep water, can you recover to surface in a lean down position (with your belly facing the bottom of the pool) to start swimming?			

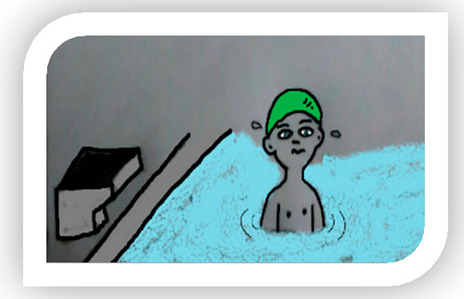
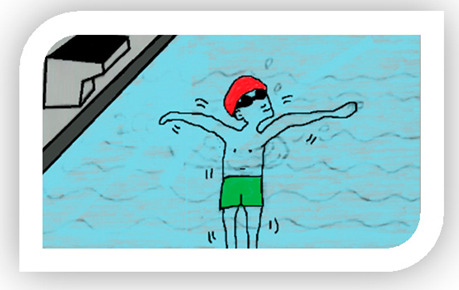
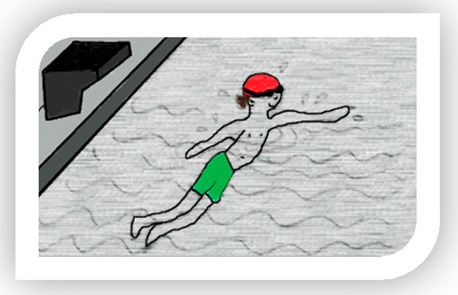
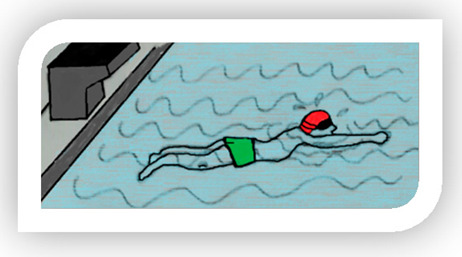
			
A. “I cannot start swimming”	B. “I can start swimming but in an upright position”	C. “I can start swimming, but my feet are in a lower position”	D. “I can start swimming with my body completely at the water surface”

(*Sk3*) If you jump into the water, can you swim underwater and through the hoops?				

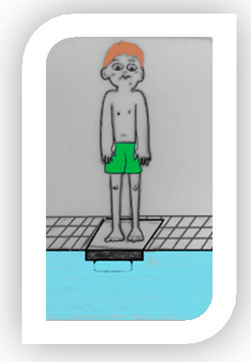
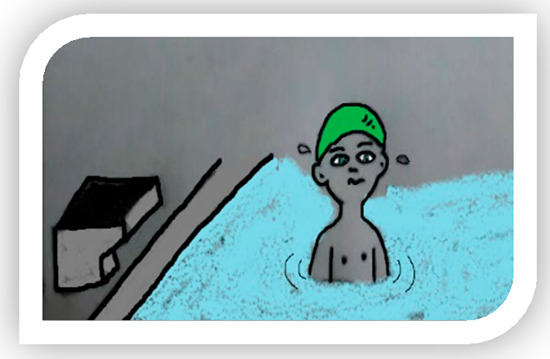
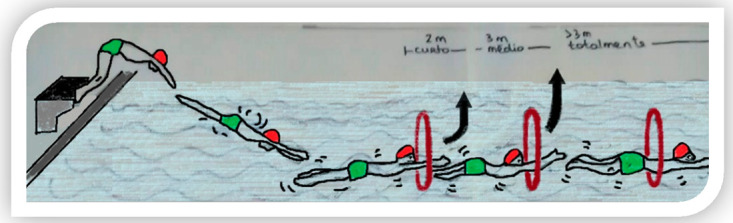
		
A. “I cannot jump into the water”	B. “I can jump into the water, but I cannot swim underwater”	C. “I can jump into the water and perform a short underwater course (through one hoop)”	D. “I can jump into the water and perform a good underwater course (through two hoops)”	E. “I can jump into the water and perform a very long underwater course (through more than two hoops)”

(*Sk4a*) Can you swim front crawl?				

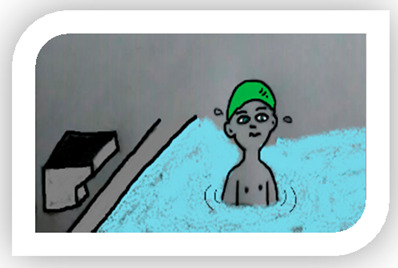
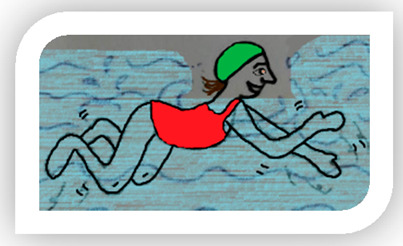
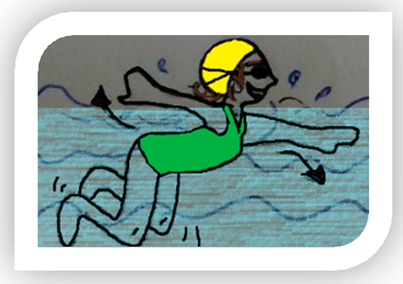
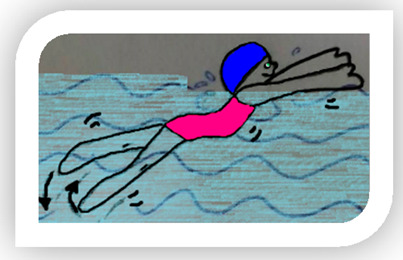
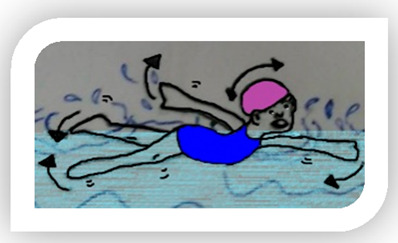
				
A. “I cannot swim”	B. “I can only swim like a dog paddle”	C. “I can swim in a tilted position, moving my arms mostly through the water. I stop swimming, or I lift my head forward to breathe,”	D. “I can swim, moving my arms and legs alternately but I have no breathing control”	E. “I can swim front crawl well, without any difficulty in breathing”

(*Sk4b*) Can you swim backstroke?				

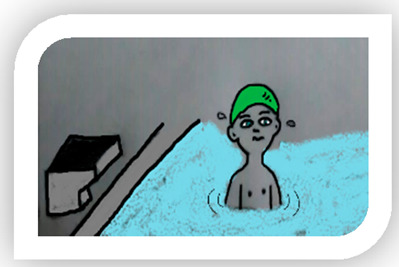
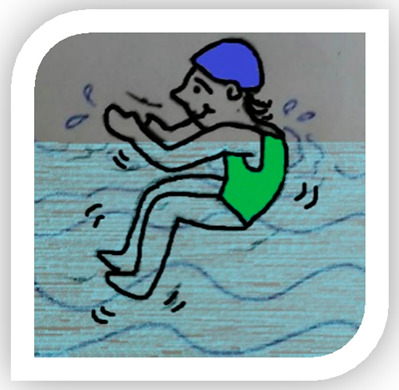
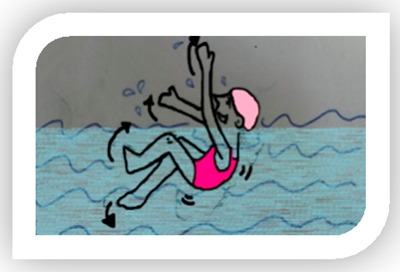
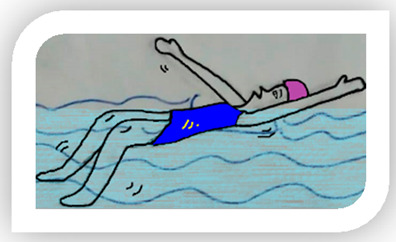
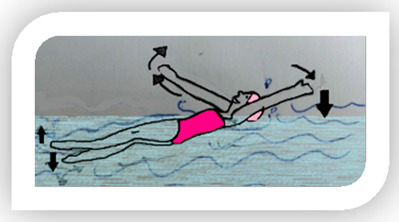
				
A. “I cannot swim”	B. “I can swim in a shrunken position (almost sitting) and move practically only the arms”	C. “I can swim in a tilted position, moving my arms and legs through the water.	D. “I can swim backstroke, with alternating movements of arms but my feet are in a lower position”	E. “I can swim backstroke well, without any difficulty”

(*Sk5*) Do you master a proper breathing technique while swimming (front crawl)?			

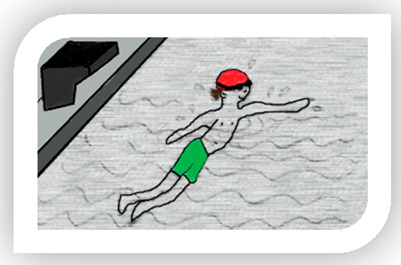
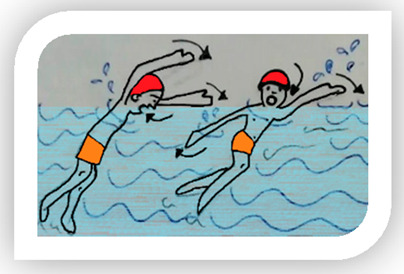
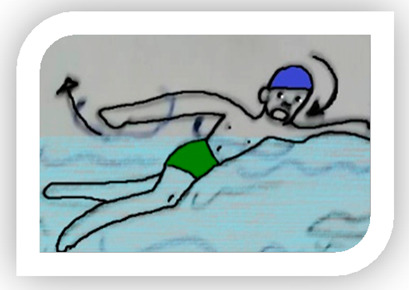
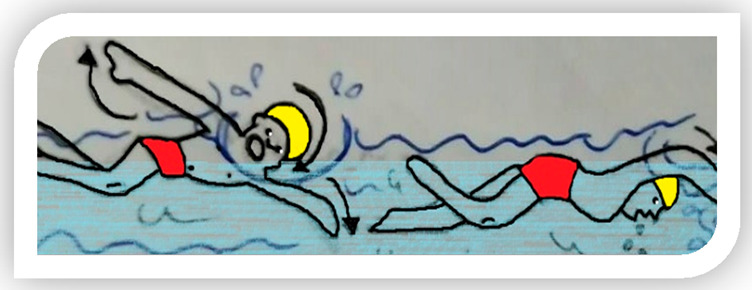
			
A. “I cannot breathe during the swim”	B. “I try to breathe while swimming, but in order to do so I stop moving my arms and legs, and I usually sink”	C. “I can breathe while swimming, but when I lift my head to breathe, I lose a lot of speed and direction”	D. “I master a good breathing technique while swimming, without any difficulty”

(*Sk6*) Can you make a body rotation in the water without any support?		

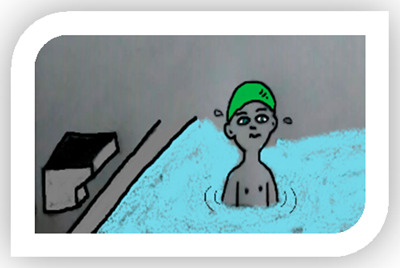
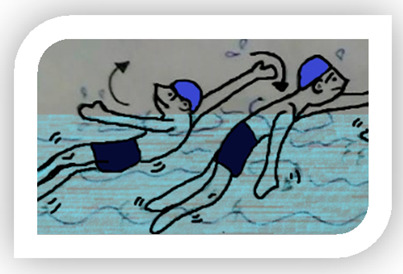
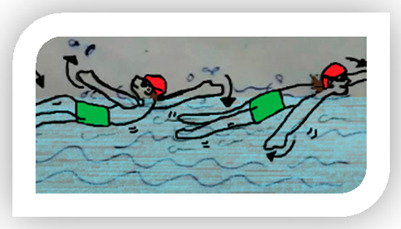
		
A. “I cannot change my body position without any support”	B. “I can do a body rotation without any support, but I usually cannot finish the rotation in the same position that I have started, so the legs tend to sink”	C. “I can do a rotation of the body in the water without any support and finish it in the same position I have started (lying in the water)”

(*Sk7a*) Can you get around the balloons while you are swimming front crawl without stopping or putting your feet on the ground?		

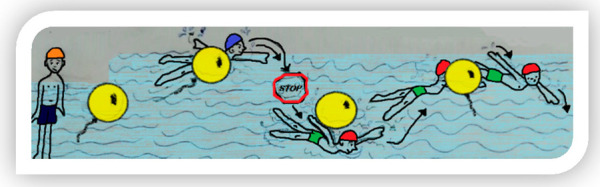

A. “I cannot get around the balloons while I swim without putting my feet on the ground.”	B. “I can, but I have to stop swimming to get around the balloons and change direction”	C. “I can contour the balloons and change direction while swimming”

(*Sk7b*) Can you get around the balloons while you are swimming backstroke without stopping or putting your feet on the ground?		

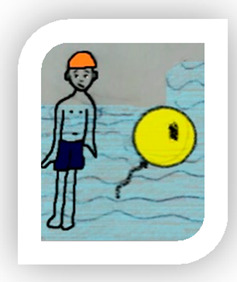
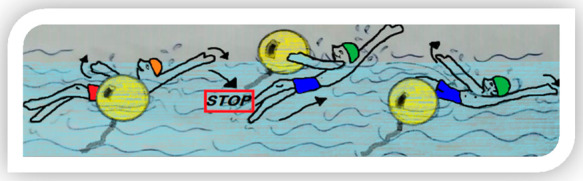
	
A. “I cannot get around the balloons while I swim backstroke without putting my feet on the ground.”	B. “I can, but I have to stop swimming to get around the balloons and change direction”	C. “I can contour the balloons and change direction while swimming backstroke”

(*Sk8*) Can you float for 30 s in a footless area without moving your arms and legs?		

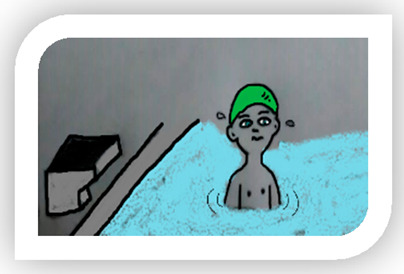
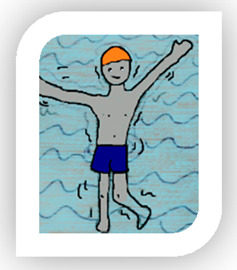
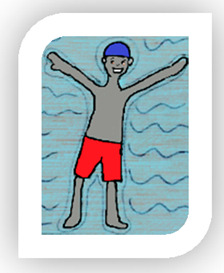
		
“I cannot float for so long”	“I can, but I have to move my arms and legs to keep myself at the surface”	“I can float very well and almost without moving”
